# The Effects of Uric Acid, Serum Vitamin D3, and Their Interaction on Parkinson's Disease Severity

**DOI:** 10.1155/2015/463483

**Published:** 2015-02-24

**Authors:** Rokhsareh Meamar, Pooria Shaabani, Seyed Reza Tabibian, Mohammad Reza Aghaye Ghazvini, Awat Feizi

**Affiliations:** ^1^Department of Medical Science, Islamic Azad University, Najafabad Branch, Isfahan 517, Iran; ^2^Isfahan Neurosciences Research Center, Isfahan University of Medical Sciences, Isfahan 8174673461, Iran; ^3^Faculty of Medicine, Isfahan University of Medical Sciences, Isfahan 8174673461, Iran; ^4^National Institute of Health Research, Tehran University of Medical Science, Tehran, Iran; ^5^Department of Biostatistics and Epidemiology, School of Health and Isfahan Endocrinology and Metabolism Research Center, Isfahan University of Medical Sciences, Isfahan 8174673461, Iran

## Abstract

*Objectives*. In current study, the relationships between serum vitamin D3 levels and serum UA concentrations as well as their interaction with severity of PD were evaluated in a sample of Iranian PD patients.* Method*. In a cross sectional study at the one of the main referral hospitals in central region of Iran, during September to November 2011, 112 patients were recruited. Severity of PD was evaluated sing H&R stages and UPDRS.* Results*. The Spearman rank correlation coefficient suggests the negative significant association between serum vitamin D3 and UPDRS in patients aged >62 (*r* = −0.34, *P* < 0.05). No statistically significant association was observed between the UA levels and severity of PD (represented by H&Y categories) in different levels of serum vitamin D3 not only in total sample but also in separate age and sex groups. The linear regression coefficients suggested positive association between UA and serum vitamin D3 with UPDRSIII scores while negative relationship between UA and serum vitamin D3 interaction with UPDRSIII; however it was only statistically significant in age group ≤62 (*P* < 0.05). *Conclusion*. Our study revealed a negative correlation between interaction of serum vitamin D3 and UA with severity of PD; other studies are required to confirm our findings.

## 1. Introduction

Parkinson disease (PD) is a common neurodegenerative disorder, characterized by motor dysfunctions including rest tremor, rigidity, and bradykinesia [1, 2] which considerably impair the quality of life of the PD patients [3].

PD was considered to be largely unknown and its pathogenic mechanisms remained uncertain. However, several genetic factors have been reported in the last decade [4, 5]. Although the eight markers' genes were significantly correlated with PD and they do not seem to be involved in a common biologic pathway or process, all are expressed in the human brain [6]. Two of the eight genes, that is, the 25-hydroxyvitamin D (vitamin D3) receptor gene (VDR) and the huntingtin interacting protein 2, have been related to PD pathophysiology and a third gene, CLTB, is involved in dopamine transporter endocytosis [6, 7]. On the other hand, it seems that the deficiency of vitamin D3 intake has a considerable influence on the pathogenesis of PD. There is evidence for abnormalities in vitamin D3 and the endocrine system in patients with PD and a higher prevalence of vitamin D3 deficiency has also been reported compared to age-matched healthy controls [8–10]. Interestingly, individuals with higher concentrations of serum vitamin D3 show a reduced risk of PD [11, 12]. Low vitamin D3 status plays an important role in PD [13]. It is reported that the distribution of vitamin D3 receptors in the substantia nigra is widely known to be affected in PD and the involvement of this vitamin has been revealed in the regulation of tyrosine hydroxylase gene expression and consequently dopamine biosynthesis [10, 14]. Some studies have identified that the level of serum vitamin D3 is also negatively interrelated with PD severity [6, 15, 16], while some others did not confirm these results [17].

Accumulating evidence confirms that oxidative stress contributes to the loss of dopaminergic neurons in the substantia nigra of PD patients [18, 19]. Uric acid (UA) is a potent endogenous antioxidant in the central nervous system [5, 18, 19]. Relevance of UA in PD pathophysiology was first suggested by the putative antioxidant properties of UA. Low levels of UA identified in the substantia nigra of PD patients and the ability of UA to decrease dopamine oxidation confirm that oxidative stress contributes to the loss of dopaminergic neurons in PD [7]. A series of epidemiological investigations explained that higher levels of UA concentration in the cerebrospinal fluid are associated with slower progression of the disease [20–23]. Moreover, serum UA levels are inversely associated with PD disease severity and duration or levodopa administration in PD patients [24, 25]. Also, Peng et al. showed that lower serum UA concentrations in men were associated with a higher PD risk [26]. In addition, a recent study in China revealed that insufficiency in serum vitamin D3 was significantly associated with elevated uric acid among postmenopausal Chinese Han women, while the precise relationship between serum vitamin D3 level and serum UA level was not recognized [27].

The relationships between UA levels as well as serum vitamin D3 with PD have been demonstrated separately; however, to our knowledge a possible link between these factors and particularly their interaction with PD severity has not been explored yet. Therefore, in the current study, the relationships between serum vitamin D3 levels and serum UA concentrations as well as their interaction with severity of PD were evaluated in a sample of Iranian PD patients.

## 2. Material and Methods

### 2.1. Study Design and Participants

Our cross-sectional, observational study was established from September to November 2011 at the Al-Zahra Hospital (affiliated to Isfahan University of Medical Sciences), one of the main referral hospitals in central region of Iran. One hundred and twelve patients diagnosed with clinically definite PD according to the clinical criteria of the UKPD Society Brain Bank [28] were recruited in this study. Patients with relatively similar pharmacological treatment were enrolled and who had recently taken UA lowering agents and vitamin D supplements (1- or 25-hydroxyvitamin D) were excluded.

A diagnosis of PD was made clinically and disease severity was judged by experienced clinical neurologist. Two major clinical scales were used: the Unified Parkinson's Disease Rating Scale (UPDRS) and Hoehn and Yahr Scale (H&Y) [29, 30]. Data about age, gender, and disease duration was also collected. We defined disease duration as the period of time between diagnosis of PD and the clinical assessment for entry into this study.

Informed written consent was obtained from each study participant and research ethic committee of Isfahan University of Medical Sciences reviewed and approved the study protocol.

### 2.2. Procedures and Variables Assessment

For each participant, fasting blood samples were obtained on the day that H&Y stage and UPDRS were evaluated. Serum was immediately separated and stored at −20C. 25-Hydroxyvitamin D levels were measured by enzyme immunoassay (Biomerica, CA, and IDS, UK) [31] and serum levels of UA were determined using Olympus AU600 random access analyzer (Olympus Corp., Japan). The commercial kits used for determining UA and serum vitamin D3 had the sensitivity of 0.3 (mg/dL) and 5 (nmol/L), respectively.

In order for analyses' proposes, we considered the following categories: uric acid level in two categories (the first category as ≤5.4 (lower than median) and the second category as >5.4 (higher than median)), age (≤62 (lower than median) and >62 (higher than median)), and H&Y (the first category: 1–1.5, the second category: 2, and the third category: >2.4).

### 2.3. Statistical Analysis

The continuous data were presented as mean ± SD and while categorical variables as frequencies (percentage). Normality of continuous data was evaluated using Kolmogorov-Smirnov test and P-P plot. Nonnormal data were subjected to logarithmic transformation. Independent samples *t*-test and Mann-Whitney *U* test were used for comparing the continuous variables with normal and nonnormal distribution between considered groups, respectively. Chi-square test was used for evaluating the association between categorical variables. Spearman rank correlation coefficient was used for assessing the bivariate association between continuous variables. Linear and logistic regression models were fitted in different models for studying the association between UPDRS scores and H&Y stages as dependent variables, respectively, and UA and serum vitamin D3 levels as well as their interaction as independent variables. In crude model, the associations between two main independent variables as well as their interaction and dependent variables were evaluated, while in other models adjustments were made for different confounding variables including age, sex, and disease duration. All mentioned models were fitted in all samples as well as in age and sex groups separately. All statistical analyses were conducted using SPSS software version 15 (SPSS Corp., Chicago, IL, USA).

## 3. Results

One hundred and twelve patients agreed to participate in our study. Mean ± SD age and disease duration were 62 ± 11.95 years and 48 ± 49.65 months, respectively. Majority of participants were male (75 (66.9%)) and the remaining were female (37 (33.1%)). More than one-third of the patients (38.4%) showed deficiency levels (<20 ng/mL) and a majority of subjects (72.8%) had insufficient levels of serum vitamin D3 (<30 ng/mL). Mean ± SD of severity values of PD, measured by UPRDSIII and H&Y stages, were 18 ± 18.21 and 2 ± 0.90, respectively.  Table 1 presents the levels of serum vitamin D3, UA, and the UPDRS score in all studied samples as well as in different age and sex groups.

The complete feature of bivariate association based on the Spearman rank correlation coefficient, between duration of the disease, serum UA, vitamin D3, and severity of the disease in total sample, as well as in different age and sex groups, has been presented in Table 2. The values and signs of the correlation coefficients demonstrate the strength and direction of the presented associations.

Table 3 shows the results of Chi-square test for evaluating the relationship between UA levels and severity of PD (represented by H&Y categories) in different levels of serum vitamin D3 (deficiency and insufficiency levels). The analysis was conducted in total study sample as well as in age and sex groups. No statistically significant association was observed between severity of PD and UA in categories of serum vitamin D3, not only in total sample but also in separate age and sex categories (the stratified analysis results based on sex and age groups have not been presented).

Table 4 represents the results of multiple linear regression analysis in different models, for total study sample as well as age and sex groups, for evaluating the association between UA, serum vitamin D3, and their interaction with UPDRSIII scores. In Model 1, the crude relationships of UA, serum vitamin D3, and their interaction with UPDRSIII were evaluated. As can be seen in this table, the regression coefficients suggest positive associations between UA and serum vitamin D3 with UPDRSIII and negative associations between interaction of UA and serum vitamin D3 with UPDRSIII. However, the obtained results were only statistically significant in age group ≤62 (*P* < 0.05). It should be noted that when adjustment was made for sex and disease duration (Models 2 and 3), we did not find any significant association.

Table 5 represents the results of multiple binary logistic regression in different models, for total study samples as well as age and sex groups, for exploring the association of H&Y (the first category: 1-2; the second category: >2.4) as dependent variable with serum UA, serum vitamin D3, and their interaction as independent variables. The presented values in this table are odds ratio (OR) and 95% confidence interval (CI) for OR. The presented ORs in the crude models show that the higher values of UA and serum vitamin D3 are associated with greater odds of being in the second category of H&Y (OR > 1), while smaller odds (OR < 1) were observed on the association of interaction of UA and vitamin D3 and being in the second category of H&Y. But as can be seen, in age group above 62 years old, the observed associations were reversed, in which the higher levels of UA and serum vitamin D3 were related inversely (OR < 1) and their interaction were directly associated (OR > 1) with odds of being in the second category of H&Y. However, it should be noted that the observed associations in crude models were reversed in some instances after taking into account potential confounding variables, including age, gender, and disease duration (Table 5, Models 2 and 3). No overall significant associations were seen between UA, serum vitamin D3, and their interaction with H&Y (Table 5).

## 4. Discussion

In this cross-sectional correlational study, the associations of serum vitamin D3, UA levels, and their interaction with severity of PD in a sample of Iranian patients were investigated. Positive associations were observed between UA and serum vitamin D3 with severity of disease when measured by UPDRS and H&Y in total study's sample and in different age and gender groups based on simple correlation analysis; however, negative significant correlation was observed between serum vitamin D3 and UPDRS in patients aged above 62 years. We did not find any significant association between serum vitamin D3, serum UA concentrations, and their interaction with UPDRS and H&Y based on multiple linear and logistic regression analysis when adjustments were made for potential confounding factors such as gender, age, and disease duration.

Continuously inadequate vitamin D status plays a major role in PD progression. In a longitudinal study in Finland, risk of PD obviously reduced with higher serum vitamin D level [14] and the latter also negatively correlated with PD severity [16, 17]. In our previous study, we did not detect any correlation between serum vitamin D3 and severity of PD [17]. However, in current study, in contrast to majority of previous studies, the results of stratified analysis based on age and sex groups showed a significant positive association between disease severity, when measured by UPDRS III, and serum vitamin D3 in male patients as well as in patients younger than 62 years. Although there are some studies with positive correlation's results in this regard [32], an explanation on the obtained unclear associations in our study is that the majority of samples are in levels of insufficiency and deficiency vitamin D3, in which no major discrepancies were observed among them in terms of serum vitamin D3 levels. A negative correlation between serum vitamin D3 levels and UPDRS III was observed in patients who are older than 62 years. It could be attributed to more deficiency of vitamin D in older age groups that leads to more disease progression. This is possible to be an example of reverse causation. Persons, particularly elderly patients with more severe PD, are less ambulatory, get less sun exposure, and subsequently have lower serum vitamin D3. Despite an abundance of correlational studies, it is unknown whether serum vitamin D3 deficiency is a cause or consequence of PD or not [33].

On the other hand, although there are well-known evidences on the association of higher serum UA levels with a significantly reduced risk of PD, along with evidence for a dose-effect relationship, implying that reduced UA concentrations may have a causal role in PD [11, 21, 22, 33], our findings did not confirm such result. We observed positive but not statistically significant correlations between UA levels and disease severity when measured based on UPDRS III and H&Y scales in comprehensive analysis based on regression models. These findings can be attributed to the mean range of UA levels in our patients which was within normal range. According to our findings, there is a positive relationship between serum UA level and the severity of disease based on linear regression analysis only when severity is measured by UPDRS in patients under 62 years. Such unexpected significant results can be attributed to the low levels of serum vitamin D3 in this age group (see Table 1)

This inconsistency between our results and other previous studies could be explained by disproportion of the H&Y scores among our study samples. Only four patients with severe scores are included and the mean H&Y stage in current research was 1.8, which is lower compared with other studies.

In addition, it is known that lifestyle factors such as dietary habits and physical activity can affect serum UA [23]. Hence, our study's conflicting results concerning the association of the progression of PD with the UA levels could be explained by the small number of participants or not considered possible confounding factors such as dietary intakes (e.g., inadequate of protein intake, as a main source of UA production, that it could be mainly attributed to low socioeconomic status of Iranian PD patient) and disease subtype or age at onset. On the other hand, a more complex relationship has been suggested by Jain et al. [23]. They reported a *U*-shaped effect, so that low UA level is accompanied by a higher PD risk, but, at higher concentrations, no further effect is observed. It could be seen that UA acts with dual antioxidant properties and it may also designate a prooxidative state under certain conditions. Regulation of this switch could be determined by the microenvironment in different compartment of human organism [13].

More recently, a growing body of literature has focused on considerable influence of parathyroid hormone on UA. Vitamin D deficiency or insufficiency leads to releasing of parathyroid hormone [18]. In the current study, high prevalence of serum vitamin D3 insufficiency (72.8%) and deficiency (38.4%) in PD patients was reported, which can be another reason for lack of prominent declination of UA in our study patients. It has been shown that intake of calcium and vitamin D significantly elevates the concentration of UA [24]. In addition, hyperuricemia can suppress 1-*α*-hydroxylase and lead to decreasing vitamin D concentration in PD [24].

Considering this point that UA was influenced by different conditions in body compartment, in the present study, we evaluated UA and serum vitamin D3 as important factors in PD and looked to the body as a whole. To the best of our knowledge, this is the first study on investigating the joint association of vitamin D and UA on disease progression in Iranian population. The observed controversial correlations in previous studies encouraged us to also investigate the interaction effect of serum vitamin D3 and UA on severity of PD. Although our study revealed a nonsignificant negative correlation between vitamin D3 and UA interaction with severity of PD, such findings need to be more investigated in larger samples of PD patients.

The present study has some potential limitations. Serum UA and vitamin D3 levels are variable and were measured just once during the study. Also, our sample was small in a cross-sectional observational study's framework; hence our findings should be interpreted cautiously. Unavailability of data on dietary intake of vitamin D and protein as well as important factors such as body mass index (BMI), medications, and genetics can be considered as another important limitation.

## Figures and Tables

**Table 1:** The mean values of uric acid level, vitamin D3 level, and UPDRS score in total study sample and in different age and sex groups.

	UA (mg/dL)	*P* value	Serum vitamin D3 (ng/mL)	*P* value	Duration (month)	*P* value	UPDRS	*P* value
Total sample	5.45 + 1.54	—	28.47 + 14.24	—	56.61 + 40.8	—	23.09 + 17.86	—
Age (year)								
≤62	5.32 ± 1.70	0.377	24.82 ± 12.32	0.008	52.22 ± 43.09	0.401	19.21 ± 18.06	0.017
>62	5.57 ± 1.38		32.14 ± 16.15		61.00 ± 38.51		26.98 ± 17.64	
Sex								
Male	5.53 ± 1.33	0.354	28.68 ± 14.57	0.604	56.16 ± 47.34	0.686	24.30 ± 18.84	0.251
Female	5.25 ± 1.94		27.10 ± 14.94		60.02 ± 54.54		20.29 ± 16.76	

UPDRS: Unified Parkinson's Disease Rating Scale III; UA: uric acid. All presented *P* value resulted from independent samples *t*-test or Mann-Whitney *U* test.

**Table 2:** The bivariate correlation between serum vitamin D3, UA levels, duration of disease and UPDRS, and Hoehn and Yahr scores in total sample and different age and sex groups.

	UPDRS & UA	UPDRS & serum vitamin D3	H&Y & UA	H&Y & serum vitamin D3	UPDRS & duration	H&Y & duration
Total population	0.148	0.036	0.203^*^	0.023	0.123	0.190
Age						
≤62	0.137	0.250	0.167	0.177	0.293^*^	0.62^*^
>62	0.114	−0.337^*^	0.239	−0.193	0.036	0.170
Sex						
Male	0.272^*^	0.033	0.340^*^	0.069	0.115	0.120
Female	−0.068	−0.004	−0.032	0.099	0.144	0.301

UPDRS: Unified Parkinson's Disease Rating Scale III; UA: uric acid; H&Y: Hoehn and Yahr. The presented values are Spearman rank correlation coefficient. ^*^The correlations are significant at *P* < 0.05.

**Table 3:** Distribution of Hoehn and Yahr levels in different levels of UA at two levels of serum vitamin D3 in total study samples.

	Hoehn and Yahr categories			*P* value^2^
	Category 1 (1–1.5)	Category 2 (2)	Category 3 (>2.4)	
Serum vitamin D3 (1)^a^				
UA^1^ (mg/dL)				
≤5.4	16 (48.5)	8 (24.2)	9 (27.3)	0.173
>5.4	10 (43.5)	2 (8.7)	11 (47.8)	
Serum vitamin D3 (2)^b^				
UA (mg/dL)				
≤5.4	12 (44.4)	9 (33.3)	6 (22.2)	0.298
>5.4	9 (31)	8 (27.6)	12 (41.4)	

^1^UA: uric acid (5.4 is the median value); ^2^resulting from Chi-square test; ^a^deficiency levels; ^b^insufficiency levels. All presented values are number (percent).

**Table 4:** The association of UPDRS scores with UA, serum vitamin D3, and their interaction in total sample as well as in age and sex groups^1^.

	Model 1	*P* value	Model 2	*P* value	Model 3	*P* value
	*B* (SE)		*B* (SE)		*B* (SE)	
Total samples						
UA (mg/dL)	0.027 (0.03)	0.31	0.03 (0.03)	0.31	0.01 (0.03)	0.65
Serum vitamin D3 (ng/mL)	0.27 (0.14)	0.06	0.27 (0.14)	0.05	0.18 (0.14)	0.22
UA & D3	−0.005 (0.004)	0.27	−0.01 (0.01)	0.28	−0.003 (0.01)	0.49
Sex^a^						
Male						
UA (mg/dL)	0.05 (0.04)	0.18	0.44 (0.24)	0.03	0.04 (0.04)	0.24
Serum vitamin D3 (ng/mL)	0.44 (0.20)	**0.03**	0.05 (0.21)	0.18	0.38 (0.21)	0.06
UA & D3	−0.01 (0.01)	0.48	0.01 (0.01)	0.48	−0.008 (0.01)	0.19
Female						
UA	0.004 (0.04)	0.93	0.08 (0.04)	0.71	−0.032 (0.03)	0.41
Serum vitamin D3 (ng/mL)	0.08 (0.20)	0.71	0.004 (0.20)	0.93	−0.07 (0.21)	0.77
UA & D3	−0.001 (0.01)	0.90	0.001 (0.01)	0.90	0.003 (0.01)	0.60
Age^b^ (year)						
≤62						
UA (mg/dL)	0.08 (0.040)	**0.04**	0.08 (0.04)	0.07	0.07 (0.04)	0.09
Serum vitamin D3 (ng/mL)	0.44 (0.18)	**0.01**	0.38 (0.18)	**0.03**	0.36 (0.19)	0.06
UA & D3	−0.02 (0.01)	**0.04**	0.01 (0.01)	0.08	−0.01 (0.01)	0.12
>62						
UA (mg/dL)	−0.05 (0.041)	0.27	0.04 (0.04)	0.31	−0.04 (0.04)	0.31
Serum vitamin D3 (ng/mL)	−0.10 (0.25)	0.69	0.07 (0.25)	0.79	−0.07 (0.25)	0.79
UA & D3	0.01 (0.01)	0.43	0.01 (0.01)	0.48	0.005 (0.01)	0.48

^1^All presented values are regression coefficient (*B*) and its standard error (SE); (Model 1) no adjustment was done for confounding variables; (Model 2) adjustment was done for age and sex; (Model 3) adjustment was done for age, sex, and diseases duration. ^a^Adjustments were made for age and disease duration in Models 2 and 3; ^b^adjustments were made for sex and disease duration in Models 2 and 3. The bold numbers are statistically significant at *P* < 0.05; UPDRS: Unified Parkinson's Disease Rating Scale III; UA: uric acid.

**Table 5:** Crude and adjusted odds ratio (OR) and 95% CI for OR of the association of H&Y with UA, serum vitamin D3, and their interaction in total study sample as well as in age and sex groups^1^.

	Model 1	Model 2	Model 3
	H&Y	H&Y	H&Y
Total samples			
UA (mg/dL)	1.41 (0.81–2.43)	1.3 (0.74–2.29)	1.31 (0.74–2.04)
Serum vitamin D3 (ng/mL)	1.01 (0.91–1.11)	0.99 (0.98–1.12)	0.99 (0.89–1.10)
UA & D3	0.99 (0.98–1.01)	0.99 (0.98–1.02)	0.99 (0.98–1.02)
			
Sex			
Male			
UA(mg/dL)	1.83 (0.82–4.06)	1.79 (0.803–4.01)	1.80 (0.79–4.01)
Serum vitamin D3 (ng/mL)	1.04 (0.91–1.20)	1.04 (0.90–1.20)	1.04 (0.90–1.20)
UA & D3	0.994 (0.97–1.02)	0.994 (0.97–1.19)	0.99 (0.97–1.02)
Female			
UA (mg/dL)	1.29 (0.55–3.03)	1.08 (0.38–3.03)	0.90 (0.19–4.29)
Serum vitamin D3 (ng/mL)	0.98 (0.82–1.16)	0.91 (0.744–1.12)	0.81 (0.57–1.16)
UA & D3	0.99 (0.97–1.03)	1.00 (0.969–1.04)	1.01 (0.95–1.05)
Age(year)			
≤62			
UA (mg/dL)	1.90 (0.95–3.80)	1.76 (0.86–3.61)	1.59 (0.75–3.36)
Serum vitamin D3 (ng/mL)	1.13 (0.97–1.32)	1.12 (0.95–1.31)	1.10 (0.94–1.29)
UA & D3	0.98 (0.96–1.01)	0.99 (0.96–1.01)	0.99 (0.96–1.01)
>62			
UA (mg/dL)	0.83 (0.32–2.16)	0.87 (0.32–2.39)	0.96 (0.34–2.72)
Serum vitamin D3 (ng/mL)	0.89 (0.76–1.05)	0.89 (0.752–1.05)	0.89 (0.76–1.06)
UA & D3	1.02 (0.99–1.04)	1.02 (0.99–1.04)	1.01 (0.98–1.04)

^1^All presented values are OR (95% CI for OR) which resulted from multivariable logistic regression; (Model 1) no adjustment was done for confounding variables; (Model 2) adjustment was done for age and sex; (Model 3) adjustment was done for age, sex, and diseases duration; H&Y: Hoehn and Yahr.
